# Prenatal tobacco exposure and ADHD symptoms at pre-school age: the Hokkaido Study on Environment and Children’s Health

**DOI:** 10.1186/s12199-019-0834-4

**Published:** 2019-12-07

**Authors:** Machiko Minatoya, Atsuko Araki, Sachiko Itoh, Keiko Yamazaki, Sumitaka Kobayashi, Chihiro Miyashita, Seiko Sasaki, Reiko Kishi

**Affiliations:** 1 Center for Environmental and Health Sciences, Hokkaido University, Kita 12, Nishi 7, Kita-ku, Sapporo, 060-0812 Japan; 2 Faculty of Health Sciences, Hokkaido University, Kita 12, Nishi 5, Kita-ku, Sapporo, 060-0812 Japan; 3 Department of Public Health Sciences, Hokkaido University Graduate School of Medicine, Kita 15, Nishi 7, Kita-ku, Sapporo, 060-8638 Japan

**Keywords:** ADHD, Prenatal tobacco exposure, Passive smoking, Birth cohort, SDQ

## Abstract

**Background:**

There have been inconsistent findings reported on maternal passive smoking during pregnancy and child risk of ADHD. In this study, ADHD symptoms at pre-school age children in association with prenatal passive and active tobacco smoke exposure determined by maternal plasma cotinine levels in the third trimester were investigated.

**Methods:**

This was a follow-up study of the birth cohort: the Hokkaido Study on Environment and Children’s Health. Children whose parents answered Strengths and Difficulties Questionnaire (SDQ) to identify child ADHD symptoms (hyperactivity/inattention and conduct problems) and total difficulties at age 5 years with available maternal plasma cotinine level at the third trimester were included (*n* = 3216). Cotinine levels were categorized into 4 groups; ≦ 0.21 ng/ml (non-smoker), 0.22–0.51 ng/ml (low-passive smoker), 0.52–11.48 ng/ml (high-passive smoker), and ≧ 11.49 ng/ml (active smoker).

**Results:**

Maternal cotinine levels of active smokers were significantly associated with an increased risk of total difficulties (OR = 1.67) and maternal low- and high-passive smoking also increased the risk (OR = 1.11, 1.25, respectively) without statistical significance. Similarly, maternal cotinine levels of active smokers were associated with an increased risk of hyperactivity/inattention (OR = 1.49). Maternal low- and high-passive smoking and active smoking increased the risk of hyperactivity/inattention (OR = 1.45, 1.43, and OR = 1.59, respectively) only in boys.

**Conclusion:**

Our findings suggested that maternal active smoking during pregnancy may contribute to the increased risk of child total difficulties and hyperactivity/inattention at pre-school age. Pregnant women should be encouraged to quit smoking and avoid exposure to tobacco smoke.

## Introduction

Children whose mothers or parents smoked during pregnancy reported to be associated with a higher risk of behavioral problems [1, 2]. Particularly, neurodevelopmental disorders including attention deficit hyperactivity disorder (ADHD) have been constantly reported to be associated with tobacco smoking exposure from maternal active smoking during pregnancy [3–5]. Health effects of maternal passive smoking have not been well studied compared to maternal active smoking during pregnancy. According to the recent review [6], inconsistent results have been found on maternal passive smoking during pregnancy in association with child ADHD symptoms. Most of the previous epidemiological studies examining maternal active and passive smoking during pregnancy only relied on a self-reported questionnaire to classify maternal smoking status. Self-reported maternal smoking during pregnancy is a feasible method that can assess exposure in an inexpensive way. However, this method tends to cause exposure misclassification such as recall bias and under-reporting or hiding of smoking because pregnant women might be aware of various adverse health effects on children who exposed to tobacco smoke during pregnancy [7, 8]. The other limitation is difficulty in quantifying tobacco smoke exposure from the environment including public and workplaces and from smokers in the same household because pregnant women may have been exposed to tobacco smoke without their notice.

Using a marker of tobacco smoke exposure such as cotinine, nicotine’s major metabolite, levels can be desirable for accurate exposure assessment, especially for passive smoke exposure assessment. There have been a few studies that used maternal cotinine levels as a marker of tobacco smoke exposure. However, the detection limit (LOD) of the cotinine level was 2 ng/ml in the previous study [9] and thus, cotinine levels below LOD but with passive smoke exposure may not be accurately assessed. The other study classified cotinine levels into only two categories (≦10 ng/ml as a nonsmoker, > 10 ng/ml as a smoker) and thus, it was not allowed to investigate the effect of passive smoking on child behavioral problems [10]. Investigation of the effects of passive tobacco smoke exposure during pregnancy on child neurobehavioral problems using cotinine levels would be a key to reveal the causal relationship.

In the present study, using data from a prospective birth cohort, we examined the association of maternal third-trimester cotinine levels and ADHD symptoms at preschool age.

## Methods

### Study population

**Fig. 1:**
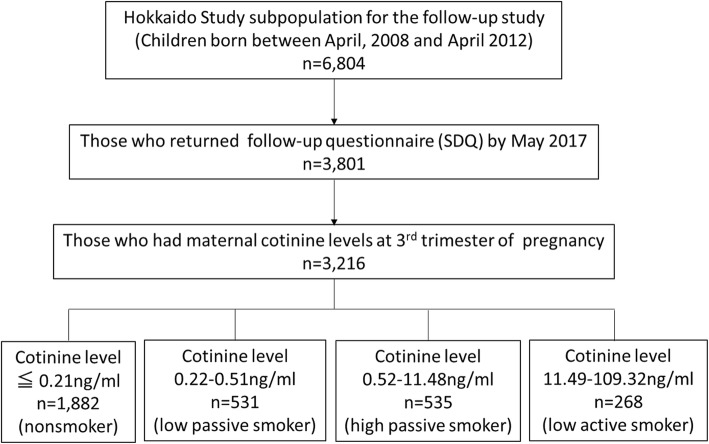
Selection of study population. Among children born between April 2008 and April 2012 (*n* = 6804), 3081 returned follow-up questionnaires by May 2017. Among 3801, maternal cotinine levels at the third trimester was available for 3216. These 3216 were categorized into four groups based on the maternal cotinine levels

This study was a follow-up study of a prospective birth cohort study, the Hokkaido Study on Environment and Children’s Health. The details of the cohort profile can be found elsewhere [11–13]. Briefly, participants were recruited during early pregnancy (< 13 weeks). The baseline questionnaire including information on demographic characteristics, smoking history, alcohol consumption, and medical history was filled by pregnant women at the recruitment. Birth weight, child sex, and diagnosis of congenital anomalies were obtained from medical records completed by obstetricians. The subpopulation consisted of cohort study participants who were born between April 2008 and April 2012 and those who have reached 5 years old were included in this study. By the end of May 2017, 3801 Strengths and Difficulties Questionnaire (SDQ) was successfully filled and returned. Among 3801 responses, those who had maternal cotinine levels in the third trimester were included in this study (*n* = 3216) (Fig. 1).

### Ethics, consent, and permissions

The protocol used in this study was approved by the Institutional Ethical Board for epidemiological studies at the Hokkaido University Graduate School of Medicine and Hokkaido University Center for Environmental and Health Sciences. This study was conducted with the informed consent of all participants in written forms.

### Exposure assessment

Maternal smoking status was examined from cotinine levels of the third-trimester maternal blood measured by using high-sensitive enzyme-linked immunosorbent assay (ELISA) developed by Cosmic Corporation, Tokyo, Japan. Blood samples were frozen at − 80 °C until assayed. Briefly, the ELISA 96-well plates coated with a rabbit anti-cotinine-4-bovine-γ-globulin polyclonal antibody were first incubated with 1% bovine serum albumin (BSA) after which 25 μL of blood plasma samples and 100 μL horseradish peroxidase-labeled (HRP) cotinine were added. The mixture was left to incubate at 20–25 °C for 1 h. Subsequent to three washes with 1% BSA, peroxidase substrate, tetramethylbenzidine, and H_2_O_2_ were added (Kirkegaad and Perry Laboratories, Gaithersburg, MD). The mixture was re-incubated for 30 min in the dark at the same temperature and 100 μL phosphoric acid was added to the wells to stop enzyme activity. The absorbance was read at a wavelength of 450 nm using an ELISA reader (Emax; Molecular Devices, Sunnyvale, CA). The LOD was 0.12 ng/ml [14]. The detailed procedure for determining cut-off values was as follows; pregnant women were categorized into three groups based on self-reported smoking status (650 smokers, 728 ex-smokers and 3750 non-smokers). Then, using the receiver operating characteristic (ROC) curve, plasma cotinine cut-off value of 11.48 ng/ml was established for separating smokers from non-smokers, resulting in a smoking prevalence of 14%. Based on our previous report which determined the cut-off levels of a nonsmoker (≦ 0.21 ng/ml), passive smoker (0.22–11.48 ng/ml), and active smoker (≧ 11.49 ng/ml) [14], we first categorized maternal cotinine levels into 3 groups. Then maternal cotinine levels in a passive smoker (*n* = 1066) were farther divided into 2 groups based on the median level (0.52 ng/ml). For the statistical analysis, 4 groups of cotinine levels; ≦ 0.21 ng/ml (nonsmoker), 0.22–0.51 ng/ml (low-passive smoker), 0.52–11.48 ng/ml (high-passive smoker), and ≧ 11.49 ng/ml (active smoker) were applied.

### Outcome measures

The SDQ was designed for a broad range of children, age 3 to 16 years and a well-validated tool of childhood mental health [15, 16]. Japanese parent-report printed version of SDQ [17] was distributed via mail to the participants. Parents were asked to fill SDQ, which included 25 items on specific strengths and difficulties with an overall rating of whether their child had behavioral problems. Each item has three response categories: (0) not true, (1) somewhat true, and (2) certainly true. It includes five subscales (conduct problems, hyperactive/inattention, emotional problems, peer problems, and prosocial behavior). All subscales excluding pro-social behavior were summed to assess the behavioral problems and the total difficulties score ranged from 0 to 40 [16]. We applied score bandings of the Japanese version of SDQ, children’s total difficulties with 0–12 were defined as normal, 13–15 were as borderline, and 16–40 were as clinical [17]. For the subscales, we used only hyperactivity/inattention and conduct problem scores to examine ADHD symptoms. The following cut-offs were applied: hyperactivity/inattention: 0–5 = normal, 6 = borderline, 7–10 = clinical; conduct problems: 0–3 = normal, 4 = borderline, 5–10 = clinical. SDQ total and subscale scores were dichotomized comparing the children with borderline and clinical scores with normal children.

### Statistical analysis

We used logistic regression models to examine maternal cotinine levels at third trimester and child ADHD symptoms at pre-school age. Covariate included in the final models was identified a priori using a directed acyclic graph: family income during pregnancy and maternal alcohol consumption during pregnancy (Additional file 1: Figure S1). In addition, we included parity and child sex in the models for all participants’ analyses based on previous literature. Paternal smoking status during pregnancy was also included to control the genetic vulnerability of both smoking and ADHD. Further analysis was conducted for stratification of child sex because the prevalence of having behavioral problems and ADHD symptoms differed between boys and girls as we previously reported [18]. Results were considered significant at two-sided *P* < 0.05. All analyses were performed by using SPSS 22.0 J (IBM Japan, Tokyo, Japan).

## Results

**Fig. 2:**
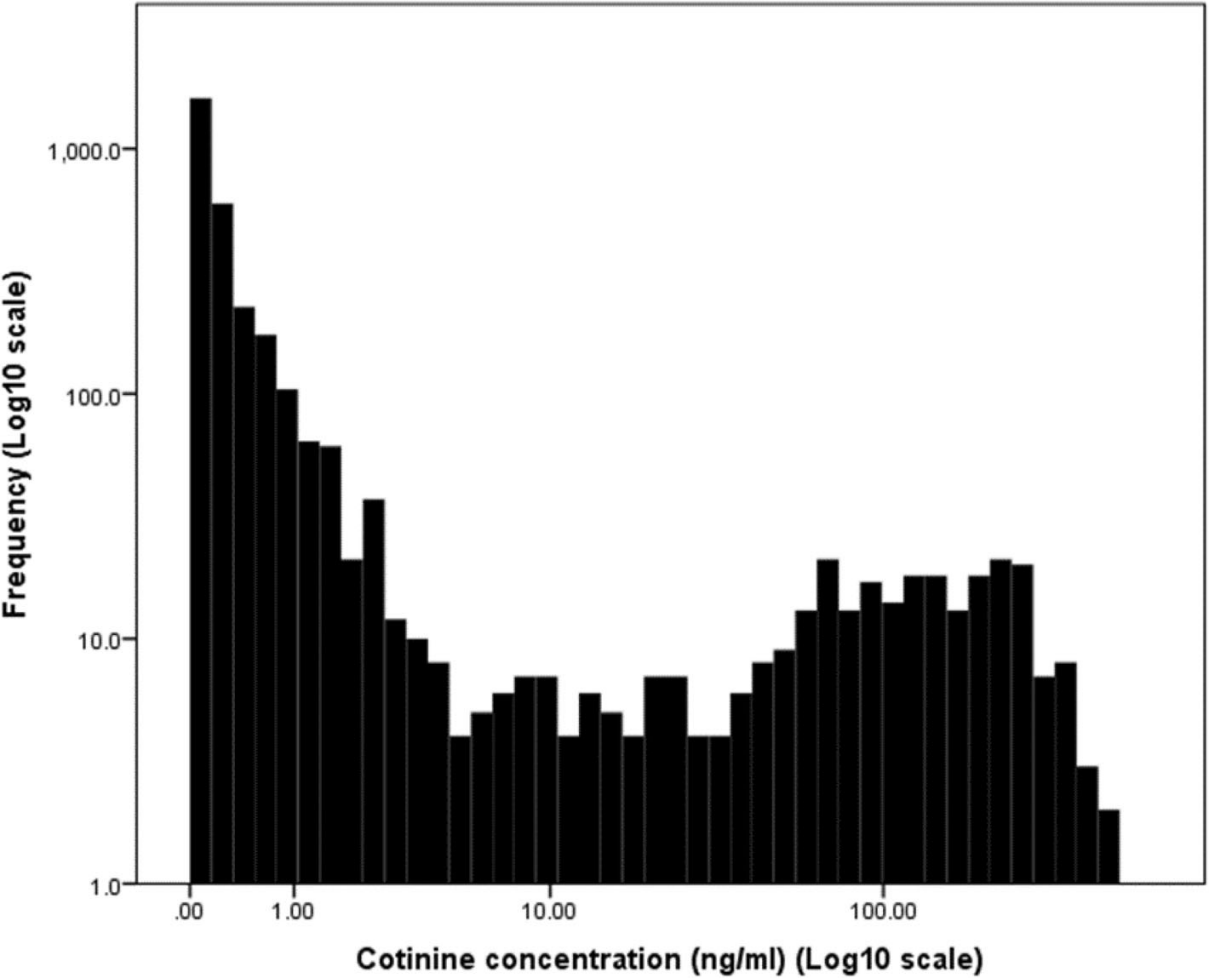
Frequency distribution of maternal cotinine levels in the third trimester. The distribution of log 10 transformed maternal cotinine levels at the third trimester was described

The characteristics of the study population according to maternal cotinine levels are shown in Table 1; 1882 (58.5%) were categorized as nonsmokers, 531 (16.5%) were categorized as low-passive smokers, 535 (16.6%) were categorized as high passive smokers, and 268 (8.3%) were categorized as smokers. Parental ages and education, parity, maternal drinking during pregnancy, family income during pregnancy and at the time of SDQ completed, and marital status were significantly different among four categories. Birth weight and length were also different among four categories and higher cotinine levels showed lower birth weight and shorter birth length. In Fig. 2, frequency distribution of maternal cotinine levels in the third trimester was shown.

**Table 1:** Characteristics of the study population according to maternal cotinine levels

Maternal cotinine (ng/ml)		All (*n* = 3216)	≦ 0.21 (*n* = 1882)	0.22-0.51 (*n* = 531)	0.52–11.48 (*n* = 535)	11.49 ≦ (*n* = 268)	*p* value
Parent							
Maternal age (years)		31.3 ± 4.7	32.0 ± 4.6	30.1 ± 4.4	30.1 ± 5.0	30.7 ± 4.9	< 0.001
Paternal age (years)		32.9 ± 5.6	33.4 ± 5.4	31.9 ± 5.2	32.2 ± 5.9	33.0 ± 5.9	< 0.001
Maternal BMI (kg/m^2^)		21.1 ± 3.2	21.2 ± 3.2	20.8 ± 2.9	21.0 ± 2.9	21.3 ± 3.6	0.083
Parity	0	1051 (32.7)	549 (29.2)	217 (40.9)	221 (39.4)	64 (23.9)	< 0.001
	≧ 1	1732 (53.9)	1086 (57.7)	240 (45.2)	241 (45.0)	165 (61.6)	
Alcohol intake during pregnancy	Yes	316 (9.8)	157 (4.9)	60 (11.3)	64 (12.0)	35 (13.1)	0.015
Maternal smoking at first trimester	Yes	185 (5.8)	9 (0.5)	4 (0.7)	14 (2.6)	158 (59.0)	< 0.001
Maternal education (years)	≦ 12	1051 (32.7)	655 (34.8)	219 (41.2)	266 (49.7)	177 (66.0)	< 0.001
	≧ 13	1732 (53.6)	1198 (63.7)	302 (56.9)	262 (49.0)	85 (31.7)	
Paternal education (years)	≦ 12	1362 (42.4)	687 (36.5)	227 (42.7)	280 (52.3)	168 (62.7)	0.001
	≧ 13	1784 (55.5)	1155 (61.4)	289 (54.4)	248 (46.4)	92 (34.3)	
Paternal smoking at first trimester	Yes	1620 (50.4%)	697 (37.5)	330 (62.7)	371 (70.4)	222 (83.5)	< 0.001
Family income during pregnancy (million JPY)	5>	1837 (57.1)	1015 (53.9)	312 (58.8)	328 (61.3)	182 (67.9)	< 0.001
	≧ 5	985 (30.6)	651 (34.6)	151 (28.4)	126 (23.6)	57 (21.3)	
Family income at SDQ completed (million JPY)	5>	1562 (48.6)	833 (44.3)	273 (51.4)	292 (54.6)	164 (61.2)	< 0.001
	≧ 5	1479 (46.0)	950 (50.5)	234 (44.1)	208 (38.9)	87 (32.5)	
Marital status	Married	3007 (93.5)	1790 (95.1)	497 (93.6)	481 (89.9)	239 (89.2)	< 0.001
Child							
Sex	Boy	1621 (50.4)	929 (49.4)	276 (52.0)	269 (50.3)	147 (54.9)	0.380
	Girl	1595 (49.6)	953 (50.6)	255 (48.0)	266 (49.7)	121 (45.1)	
Birth weight (g)		3048 ± 385	3060 ± 375	3041 ± 405	3060 ± 391	2960 ± 393	0.001
Birth length (cm)		49.0 ± 2.0	49.1 ± 1.9	49.1 ± 2.1	49.0 ± 2.0	48.7 ± 2.0	0.024

Mean ± SD or number (%). *JPY* Japanese Yen. Chi-square test or Mann Whitney’s test

Table 2 describes the number and percentage of children with behavioral problems and ADHD symptoms by child sex and maternal cotinine levels in the third trimester. Percentage of children in borderline and clinical range scores of total difficulties and ADHD symptoms were higher in boys compared to girls. Compared to cotinine levels nonsmokers, percentages of children in borderline and clinical range scores of behavioral problems and ADHD symptoms were higher in both low- and high-passive and active smokers. The more detailed distribution of child behavioral problems and ADHD symptoms according to maternal cotinine levels were shown in Additional file 1: Table S3.

**Table 2:** Number and percentage of children in borderline and clinical range scores of behavioral problems and ADHD symptoms by child sex and maternal cotinine levels at the third trimester (*n* = 3202)

		Number of children	Total difficulties	Hyperactivity/inattention	Conduct problems
Child sex	All	3216	649 (20.2)	480 (14.9)	628 (19.5)
	Boys	1621	393 (24.2)	316 (19.5)	358 (22.1)
	Girls	1595	256 (16.1)	164 (10.3)	270 (16.9)
Maternal cotinine levels (ng/ml)	≦ 0.21 (non)	1882	339 (18.0)	234 (12.4)	340 (18.1)
	0.22–0.51 (low passive)	531	112 (21.1)	97 (18.3)	107 (20.2)
	0.52–11.48 (high passive)	535	126 (23.6)	97 (18.1)	125 (23.4)
	11.49 ≧ (active)	268	72 (26.9)	52 (19.4)	56 (20.9)

*n* (%)

Table 3 shows the association between maternal cotinine levels at third trimester and child behavioral problems and ADHD symptoms. Maternal cotinine levels of active smokers were significantly associated with an increased risk of total difficulties (odds ratio (OR) = 1.67, 95% confidence interval (CI) 1.17, 2.39) and of hyperactivity/inattention (OR = 1.49, 95% CI 1.00, 2.23). Maternal cotinine levels of low- and high-passive smokers were associated with an increased risk of hyperactivity/inattention without significance (OR = 1.23, 95% CI 0.89, 1.69, OR = 1.15, 95% CI 0.82, 1.60, respectively). *P* for trend found to be significant for total difficulties (*P*_trend_ = 0.004) and marginal for hyperactivity/inattention (*P*_trend_ = 0.051). An increased risk of total difficulties in association with maternal cotinine levels of active smokers (OR = 1.94, 95% CI 1.22, 3.07) was found only in boys. An increased risk of hyperactivity/inattention was found in association with maternal cotinine levels of low- and high-passive and active smokers (OR = 1.45, 95% CI 0.97, 2.16, OR = 1.43, 95% CI 0.95, 2.17, OR = 1.59, 95% CI 0.97, 2.63, respectively) only in boys. *P* for trend for total difficulties was 0.007, for hyperactivity/inattention was 0.024, respectively among boys. Further, statistical effect modification by sex was investigated using interaction terms; however, no statistical significance was found.

**Table 3:** Association between maternal cotinine levels at the third trimester and child behavioral problems and ADHD symptoms

	Maternal cotinine (ng/ml)	Number of children	Total difficulties	Hyperactivity/inattention	Conduct problems
			OR (95% CI)		
All	≦ 0.21 (non)	1882	Reference	Reference	Reference
	0.22–0.51 (low passive)	531	1.11 (0.83, 1.49)	1.23 (0.89, 1.69)	1.07 (0.81, 1.43)
	0.52–11.48 (high passive)	535	1.25 (0.93, 1.68)	1.15 (0.82, 1.60)	1.30 (0.98, 1.72)
	11.49 ≧ (active)	268	1.67 (1.17, 2.39)*	1.49 (1.00, 2.23)^+^	1.07 (0.74, 1.56)
	*P* for trend		0.004	0.051	0.208
Boys	≦ 0.21 (non)	929	Reference	Reference	Reference
	0.22–0.51 (low passive)	276	1.36 (0.93, 1.98)	1.45 (0.97, 2.16)^+^	1.18 (0.80, 1.74)
	0.52–11.48 (high passive)	269	1.28 (0.86, 1.90)	1.43 (0.95, 2.17)^+^	1.45 (0.99, 2.14)
	11.49 ≧ (active)	140	1.94 (1.22, 3.07)*	1.59 (0.97, 2.63)^+^	1.19 (0.73, 1.95)
	P for trend		0.007	0.024	0.126
Girls	≦ 0.21 (non)	953	Reference	Reference	Reference
	0.22-0.51 (low passive)	255	0.82 (0.51, 1.34)	0.90 (0.51, 1.59)	0.95 (0.62, 1.47)
	0.52-11.48 (high passive)	266	1.23 (0.79, 1.92)	0.77 (0.42, 1.40)	1.15 (0.76, 1.76)
	11.49 ≧ (active)	121	1.36 (0.77, 2.42)	1.36 (0.69, 2.65)	0.94 (0.52, 1.67)
	*P* for trend		0.205	0.858	0.844

Adjusted for family income during pregnancy, maternal alcohol consumption during pregnancy, parity, paternal smoking status during pregnancy, and child sex (only for all)

*p < 0.05

^+^*p* < 0.10

## Discussion

In this study, we found that not only maternal active smoking but also maternal passive smoking may possibly be associated with increased risks of total difficulties and hyperactivity/inattention and the weak association was only found in boys. The significance remained after adjustments with social factors suggested that tobacco exposure during pregnancy was an independent risk factor for child behavioral problems and ADHD symptoms at pre-school age even at passive smoke exposure.

Cotinine is a direct metabolite of nicotine that has a high specificity for tobacco smoke exposure [19] even though it only indicates the amount of nicotine exposure over the past 3 days [20], the half-life is longer compared to the parental compound, nicotine which has 2 h of half-life on average [21]. Additionally, by using the cut-off of cotinine levels for active and passive smokers [14], we were able to show passive tobacco smoke exposure had significant adverse effects on child ADHD symptoms.

The smoking rate of the adult population was highest (24.7%) in Hokkaido prefecture compared to the others [22]. Compared to the Organization for Economic Cooperation and Development (OECD) data in 2017, the smoking rate of the adult population in Hokkaido is higher than OECD 34 countries average (18.4%) [23]. The smoking rate among the female population was highest (16.1%) among 47 prefectures in Japan. Accordingly, pregnant women in Hokkaido are considered to be a relatively higher risk of active and passive tobacco smoke exposure compared to the other prefectures in Japan. Based on the third-trimester maternal cotinine levels, the prevalence of maternal active smoking was 8.2% in this study, which was considered to be similar range to the European Perinatal Health Report from 2010 that estimated above 10% prevalence with a variation of under 5 to 19% [24].

Chances of quitting smoking are lower in mothers with lower social status, a smoking partner, and higher parity [25], and this trend was observed in this study. Among 185 women who answered as a smoker in the first trimester, 158 women remained to have cotinine levels as active smokers at the third trimester indicated that only 27 women (14.6%) considered to quit smoking between the first and the third trimester (Table 1). According to the review article, a wide range of smoking cessation rates during pregnancy was reported with 27–47% in Europe, 23–43% in the USA, 62–70% in Japan, and 4–47% in other countries [25]. The cessation rate of this study population, 14.6% was lower compared to the review.

About one in three pregnant women (34.6%) who did not smoke at the first trimester showed cotinine levels of passive or active smokers at the third trimester indicated that they had tobacco smoke exposure during pregnancy even though they did not smoke, and moreover, 3.5% of those who did not smoke at the first trimester showed smoker level cotinine at the third trimester indicated that they had excessive tobacco smoke exposure from other smokers during pregnancy.

For effect of maternal active smoking during pregnancy on child ADHD symptoms, our findings were in line with the previous reports included in the recent meta-analysis of 27 articles that showed prenatal exposure to maternal smoking during pregnancy was significantly associated with childhood ADHD after adjusting for parental psychiatric history and socioeconomic status, contrary, inconsistent results on the risk of maternal passive smoking during pregnancy were reported [6]. A previous article mentioned that biomarker verification along with questionnaire data may be required for passive smoking assessment [26]. A review article suggested that available cut-off value for adult pregnant populations is not applicable to the general population [27]. It was mentioned that generalization of cut-off values based only on white populations may not be appropriate for other racial/ethnic groups. In this study, we used cut-off values based on pregnant women in the Japanese population [14], which enabled us to provide more accurate evidence.

It is possible that exposure to tobacco smoking may disrupt normal brain development as it is known that nicotine impacts serotonin and dopaminergic systems, brain cell growth, DNA, and RNA synthesis of the fetal brain [28]. Findings from the animal study supported the notion that prenatal nicotine exposure induces neurobehavioral abnormalities in mouse offspring by disrupting the dopaminergic system and improved our understanding about the incidence of ADHD in children whose mothers were exposed to nicotine during their pregnancy [29]. In this study, we observed that higher maternal third trimester cotinine level was strongly associated with an increased risk of behavioral problems and hyperactivity/inattention in dose-dependent manner, which supports nicotine-related biological mechanism. Maternal use of nicotine replacement without smoking during pregnancy was associated with a higher risk of ADHD, which suggested that nicotine affects ADHD development as well [4].

Sex-specific effects of maternal smoking during pregnancy on various health outcomes neurodevelopment [30] and visual and auditory development [31] have been reported. In these previous reports, males were found to be more vulnerable to tobacco smoke exposure during pregnancy than girls. Our results were consistent with these previous findings. The recent review concluded that the brain in males is more vulnerable to many toxic exposures than it is in females [32]. Together with vulnerability to toxic exposure in boys reported previously, not only cotinine but also exposure to tobacco smoke with a complex mixture of chemicals may attribute to non-significant increased risk in boys found in this study.

Inherited effect rather than the prenatal environmental effect on childhood ADHD has been discussed [33, 34]. Heritability of both smoking and ADHD and the recent reports of genetic overlap between smoking and ADHD may indicate that maternal smoking during pregnancy may reflect a genetic predisposition rather than a causal risk factor for offspring ADHD [35]. The current study included passive smokers who supposedly not sharing a genetic overlap between smoking habit and ADHD and found that maternal passive smoking could increase the risk of childhood ADHD symptoms after controlling for the paternal smoking habit. This may indicate that tobacco smoke exposure during pregnancy is potentially associated with child ADHD risk.

We should note the limitations of this study. First, the response rate of this follow-up study was 55.9%, and this may be linked to represent a higher socioeconomic status population in this study than the whole cohort population (Additional file 1: Table S1). Since parental education and family income during pregnancy inversely correlated with SDQ scores and maternal smoking during pregnancy was positively associated with SDQ scores, our findings from the relatively higher socioeconomic population than the original cohort population might have underestimated the effects of prenatal tobacco smoking exposure on child behavioral problems and ADHD symptoms. In general, a subpopulation that is relatively healthy, wealthy remains in the follow-up in cohort studies. However, studies have demonstrated that this causes only minimal bias [36]. Second, even though we used widely used questionnaires to assess child behavioral problems and ADHD symptoms, these symptoms were reported by children’s mothers and could be affected by maternal mental disorder or mood, which we did not have information for. In addition, a parental history of hyperactivity/inattention was not obtained. Without controlling for genetic factors including maternal mental disorder and parental history of ADHD may have affected the findings from this study. Finally, postnatal exposure to tobacco smoking was not considered in this study. As shown in the recent study from Japan, parental smoke, and SES were significantly associated with low academic performance among Japanese children [37], it would have been desirable to take postnatal factors into account. However, almost every developing organ system including brain and nerve system appears to be affected by prenatal exposure to tobacco smoking. Besides, it is difficult to distinguish contributions of tobacco smoking on child ADHD symptoms from prenatal versus postnatal exposure because often times smoking habit before/during and after pregnancy remains consistent in most populations. This prevents precise analysis of the impact of tobacco smoking exposure during pregnancy and the postnatal period. In addition, it should be mentioned that electronic cigarette (e-cigarette) use in pregnancy has been steadily increasing [38]. Though e-cigarette was not popular from 2008 to 2010 in Japan, the use of e-cigarette in Japan is slightly increasing [39]. Scientific evidence regarding the human health effects of e-cigarettes is limited. While e-cigarette aerosol may contain fewer toxicants than cigarette smoke, studies evaluating whether e-cigarettes are less harmful than cigarettes are inconclusive [40]. According to the recent review, the nicotine consumed by e-cigarettes is similar to that consumed by cigarette smoking [38]. There are urgent needs for further studies considering the health impacts of both conventional tobacco smoke and emerging e-cigarette exposure on child neurodevelopment.

## Conclusion

Our findings suggested that maternal active smoking during pregnancy may contribute to the increased risk of child total difficulties and hyperactivity/inattention at pre-school age. Pregnant women should be encouraged to quit smoking and avoid exposure to tobacco smoke.

## Supplementary information

**Additional file 1: Table S1.** Comparison of characteristics in this study population (n=3,216) and in the whole follow-up population (n=6,804). **Table S2.** Correlation coefficients between characteristics of participants and SDQ scores. **Table S3.** Cotinine levels and rate of children with ADHD symptoms. Figure S1 DAG for selecting covariates.

## Data Availability

Please contact the author for data requests.

## Abbreviations

ADHD: Attention deficit hyperactivity disorder
BSA: Bovine serum albumin
CI: Confidence interval
e-cigarette: Electronic cigarette
ELISA: Enzyme-linked immunosorbent assay
HRP: Horseradish peroxidase-labeled
LOD: Limit of detection
OECD: Organization for Economic Cooperation and Development
OR: Odds ratio
ROC: Receiver operating characteristic
SDQ: Strengths and Difficulties Questionnaire
