# Supplementary material for: Prenatal tobacco exposure and ADHD symptoms at pre-school age: the Hokkaido Study on Environment and Children’s Health
Source: Environ Health Prev Med. 2019 Dec 7;24:74. doi: 10.1186/s12199-019-0834-4 (PMC6898952; doi:10.1186/s12199-019-0834-4)
Supplement: Supplementary file 1 — Additional file 1: Table S1. Comparison of characteristics in this study population (n=3,216) and in the whole follow-up population (n=6,804). Table S2. Correlation coefficients between characteristics of participants and SDQ scores. Table S3. Cotinine levels and rate of children with ADHD symptoms. Figure S1 DAG for selecting covariates. [file 12199_2019_834_MOESM1_ESM.docx]

Table S1 Comparison of characteristics in this study population (n=3,216) and in the whole follow-up population (n=6,804).

| Characteristics |  | This study population (n=3,216) | Whole follow-up population (n=6,804) |
| --- | --- | --- | --- |
| Parent |  |  |  |
| Maternal age (years) |  | 31.3 ± 4.7 | 30.8 ± 5.0 |
| Paternal age (years) |  | 32.9 ± 5.6 | 32.6 ± 5.8 |
| Maternal pre-pregnancy BMI (kg/m^2^) |  | 21.1 ± 3.1 | 21.2 ± 3.3 |
| Parity | 0 | 1,055 (32.8) | 2,205 (32.4) |
|  | ≧ 1 | 1,733 (53.8) | 3,663 (53.8) |
|  | Missing | 424 (13.2) | 936 (13.8) |
| Alcohol intake during pregnancy | Yes | 317 (9.8) | 658 (9.7) |
|  | Missing | 96 (3.0) | 296 (4.4) |
| Maternal cotinine levels at 3^rd^ trimester (ng/ml) | ≦ 0.21 | 1,882 (58.5) | 2,913 (42.8) |
|  | 0.22-11.48 | 1,066 (33.1) | 1,936 (28.5) |
|  | ≧ 11.49 | 268 (8.3) | 755 (11.1) |
|  | Missing | - | 1,200 (17.6) |
| Maternal education (years) | ≦ 12 | 1,319 (41.0) | 3,146 (46.2) |
|  | ≧ 13 | 1,820 (56.5) | 3,498 (51.4) |
|  | Missing | 77 (2.3) | 166 (2.4) |
| Paternal education (years) | ≦ 12 | 1,364 (42.3) | 3,140 (46.1) |
|  | ≧ 13 | 1,787 (55.5) | 3,423 (50.3) |
|  | Missing | 65 (2.0) | 239 (3.5) |
| Family income during pregnancy (million JPY) | < 5 | 1,840 (57.1) | 3,902 (57.3) |
|  | ≧ 5 | 987 (30.6) | 1,896 (27.9) |
|  | Missing | 389 (12.1) | 1,006 (14.8) |
| Child |  |  |  |
| Sex | Boy | 1,621 (50.4) | 3,403 (50.0) |
|  | Girl | 1,595 (49.6) | 3,401 (50.0) |
| Birth weight (g) |  | 3048 ± 385 | 3013 ± 438 |
| Birth length (cm) |  | 49.0 ± 2.0 | 48.9 ± 2.2 |

Mean ± SD or number (%). JPY: Japanese Yen.

Table S2 Correlation coefficients between characteristics of participants and SDQ scores.

| Characteristics | Total difficulties | Hyperactivity/inattention | Conduct problems |
| --- | --- | --- | --- |
| Maternal age (years) | -0.086** | -0.092** | -0.080** |
| Paternal age (years) | -0.058** | -0.042* | -0.057** |
| Maternal pre-pregnancy BMI (kg/m^2^) | 0.030 | 0.025 | 0.039* |
| Parity | -0.104** | -0.086** | -0.014 |
| Alcohol intake during pregnancy | 0.040* | 0.039* | 0.036* |
| Maternal education (years) | -0.086** | -0.075** | -0.072** |
| Paternal education (years) | -0.032 | -0.040* | -0.034 |
| Family income during pregnancy (million JPY) | -0.108** | -0.089** | -0.091** |
| Family income at SDQ completed (million JPY) | -0.105** | -0.082** | -0.100** |
| Marital status | -0.069** | -0.055** | -0.086** |
| Child Sex |  |  |  |
| Birth weight (g) | -0.008 | -0.009 | 0.007 |
| Birth length (cm) | -0.012 | -0.009 | 0.002 |

Spearman’s rho. * p < 0.050. ** p < 0.001. JPY: Japanese Yen.

Table S3 Cotinine levels and rate of children with ADHD symptoms.

| Cotinine levels (mg/ml) | All (n=3,216) | Total difficulties (n=649) | Hypertension/Inattention (n=480) | Conduct problems (n=628) |
| --- | --- | --- | --- | --- |
| LOD to < 0.20 | 1,805 | 327 (18.1) | 228 (12.6) | 331 (18.3) |
| 0.20 to < 0.30 | 317 | 67 (21.1) | 51 (16.1) | 58 (18.3) |
| 0.30 to < 0.40 | 158 | 26 (16.5) | 25 (15.8) | 32 (20.3) |
| 0.40 to < 0.50 | 107 | 26 (24.3) | 24 (22.4) | 21 (19.6) |
| 0.50 to < 0.60 | 95 | 16 (16.8) | 14 (14.7) | 16 (16.8) |
| 0.60 to < 0.70 | 61 | 10 (16.4) | 10 (16.4) | 7 (11.5) |
| 0.70 to < 0.80 | 63 | 23 (36.5) | 14 (22.2) | 18 (28.6) |
| 0.80 to < 0.90 | 40 | 8 (20.0) | 7 (17.5) | 7 (17.5) |
| 0.90 to < 1.0 | 40 | 15 (37.5) | 14 (35.0) | 13 (32.5) |
| 1.0 to < 2.0 | 153 | 37 (24.2) | 23 (15.0) | 45 (29.4) |
| 2.0 to < 3.0 | 53 | 14 (26.4) | 14 (26.4) | 14 (26.4) |
| 3.0 to < 4.0 | 18 | 3 (16.7) | 3 (16.7) | 4 (22.2) |
| 4.0 to < 5.0 | 8 | 0 (0) | 0 (0) | 0 (0) |
| 5.0 to < 6.0 | 6 | 0 (0) | 0 (0) | 2 (33.3) |
| 6.0 to < 7.0 | 2 | 0 (0) | 0 (0) | 1 (50.0) |
| 7.0 to < 8.0 | 6 | 0 (0) | 0 (0) | 0 (0) |
| 8.0 to < 9.0 | 6 | 2 (33.3) | 0 (0) | 0 (0) |
| 9.0 to < 10.0 | 3 | 1 (33.3) | 0 (0) | 0 (0) |
| 10.0 to < 20.0 | 24 | 9 (37.5) | 6 (25.0) | 8 (33.3) |
| 20.0 to <30.0 | 17 | 3 (17.6) | 4 (23.5) | 5 (29.4) |
| 30.0 to < 40.0 | 8 | 3 (37.5) | 3 (37.5) | 2 (25.0) |
| 40.0 to < 50.0 | 13 | 6 (46.2) | 1 (7.7) | 2 (15.4) |
| 50.0 to < 60.0 | 12 | 1 (8.3) | 0 (0) | 1 (8.3) |
| 60.0 to < 70.0 | 22 | 6 (27.3) | 4 (18.2) | 2 (9.1) |
| 70.0 to < 80.0 | 16 | 4 (25.0) | 4 (25.0) | 3 (18.8) |
| 80.0 to < 90.0 | 10 | 1 (10.0) | 1 (10.0) | 2 (20.0) |
| 90.0 to < 100.0 | 11 | 3 (27.3) | 2 (18.2) | 3 (27.3) |
| 100.0 to < 200.0 | 77 | 20 (26.0) | 15 (19.5) | 16 (20.8) |
| 200.0 to < 300.0 | 47 | 13 (27.7) | 8 (17.0) | 8 (17.0) |
| 300.0 to < 400.0 | 14 | 3 (21.4) | 4 (28.6) | 4 (28.6) |
| 400.0 to < 500.0 | 4 | 2 (50.0) | 1 (25.0) | 3 (75.0) |
| n (%). | | | | |


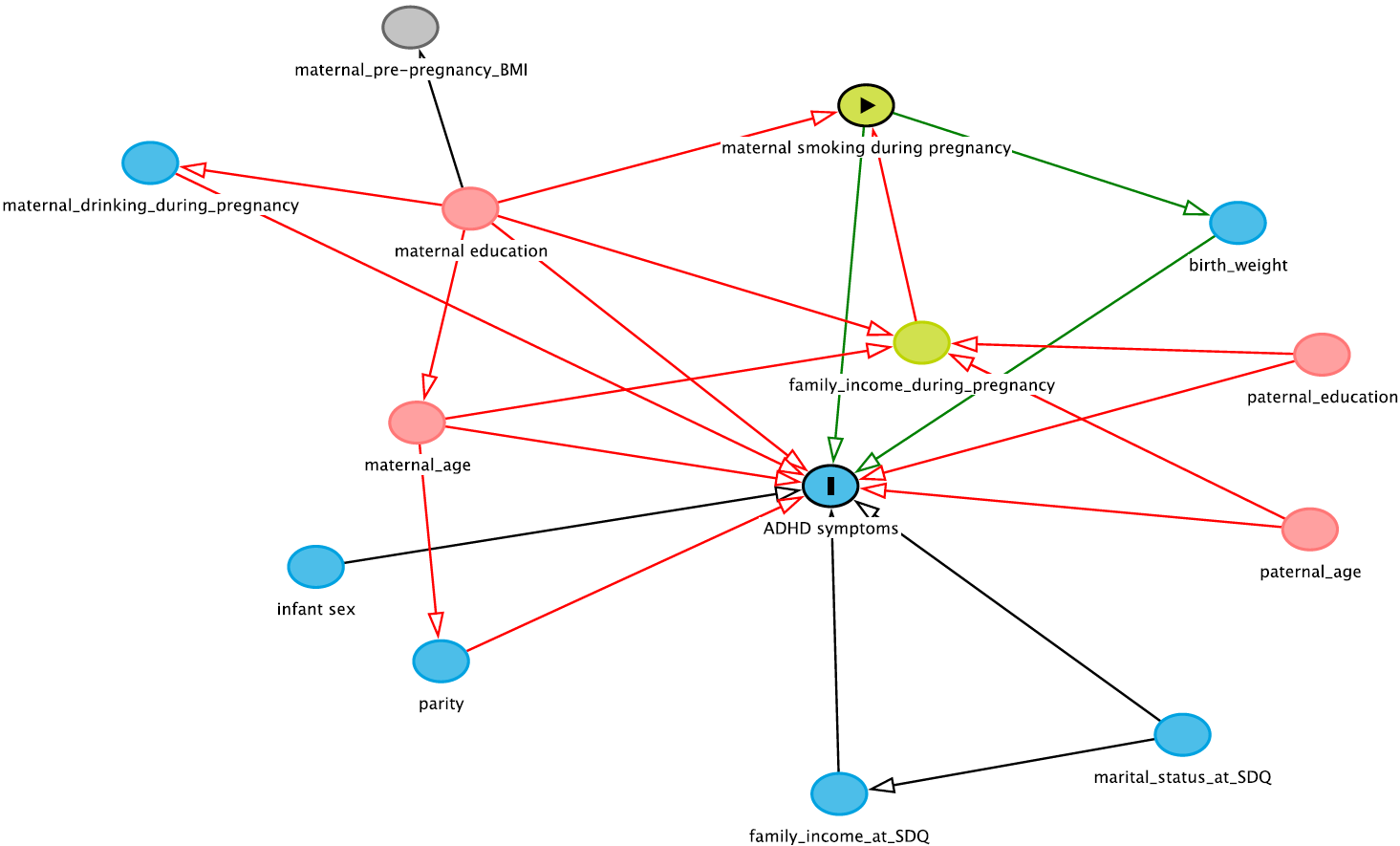


Figure S1 DAG for selecting covariates.
